# The N-Formyl Peptide Receptors and Rheumatoid Arthritis: A Dangerous Liaison or Confusing Relationship?

**DOI:** 10.3389/fimmu.2021.685214

**Published:** 2021-06-18

**Authors:** Ilaria Mormile, Francesca Wanda Rossi, Nella Prevete, Francescopaolo Granata, Valentina Pucino, Amato de Paulis

**Affiliations:** ^1^ Department of Translational Medical Sciences, University of Naples Federico II, Naples, Italy; ^2^ Center for Basic and Clinical Immunology Research (CISI), World Allergy Organization (WAO) Center of Excellence, University of Naples Federico II, Naples, Italy; ^3^ Institute of Experimental Endocrinology and Oncology (IEOS), National Research Council (CNR), Naples, Italy; ^4^ College of Medical and Dental Sciences, Institute of Inflammation and Ageing, University of Birmingham, Birmingham, United Kingdom

**Keywords:** rheumatoid arthritis, formylpeptide receptors, rheumatoid arthritis histopathotypes, pattern recognition receptors, innate immunity

## Abstract

Rheumatoid arthritis (RA) is a chronic autoimmune disease characterized by a progressive symmetric inflammation of the joints resulting in bone erosion and cartilage destruction with a progressive loss of function and joint deformity. An increased number of findings support the role of innate immunity in RA: many innate immune mechanisms are responsible for producing several cytokines and chemokines involved in RA pathogenesis, such as Tumor Necrosis Factor (TNF)-α, interleukin (IL)-6, and IL-1. Pattern recognition receptors (PRRs) play a crucial role in modulating the activity of the innate arm of the immune response. We focused our attention over the years on the expression and functions of a specific class of PRR, namely formyl peptide receptors (FPRs), which exert a key function in both sustaining and resolving the inflammatory response, depending on the context and/or the agonist. We performed a broad review of the data available in the literature on the role of FPRs and their ligands in RA. Furthermore, we queried a publicly available database collecting data from 90 RA patients with different clinic features to evaluate the possible association between FPRs and clinic-pathologic parameters of RA patients.

## Introduction

Rheumatoid arthritis (RA) is a chronic autoimmune disease characterized by a progressive symmetric inflammation of the joints resulting in bone erosion and cartilage destruction with a progressive loss of function and joint deformity (1). The major clinical characteristic of RA is joint swelling reflecting inflammation in the synovial membrane (2). Extra-articular symptoms such as pulmonary manifestations (e.g., lung nodules, pleural effusion, and interstitial lung disease), vasculitis, keratoconjunctivitis, hematological abnormalities (e.g., anemia, leukopenia, thrombocytopenia, or thrombocytosis), rheumatic nodules, and lymphomas are also possible, especially in later stages of the disease (1, 3, 4). It has been hypothesized that RA likely occurs in genetically predisposed subjects due to a combination of genetic, epigenetic, and environmental factors initiated by a stochastic event such as an infection or tissue injury (2). These triggering factors may activate the previously generated autoreactive B and T cells leading to a disruptive tolerance resulting in tissue damage (1). The tissue destruction presents as inflammation of the joint capsule (synovitis) with the expansion of the synovial membrane (pannus) that may lead to periarticular bone erosions and cartilage degradation. This chronic joint inflammation is promoted and maintained by several different cell type; the cellular composition of RA synovitis includes features of both innate (e.g., monocytes, dendritic cells (DCs), mast cells, and innate lymphoid cells) and adaptive (e.g., T helper cell (Th) 1, Th17, B cells, plasmablasts, and plasma cells) immunity, together with fibroblasts, and osteoclast (2). Hence, in RA inflammatory process, both innate and adaptive immunity are pivotal pathogenetic actors. An increased number of findings supports the role of innate immunity in RA; indeed, many innate immune mechanisms are responsible for the production of a significant proportion of cytokine and chemokine synthesis involved in RA pathogenesis, such as Tumor Necrosis Factor α (TNFα), interleukin (IL)-6, and IL-1 (5, 6). In addition, macrophage numbers and the detection of TNFα in the synovial tissue of patients with RA are good predictors of the clinical course of the disease (7) and anti-cytokine therapy effectiveness. There has been a longstanding hypothesis that infection plays a role in triggering pathways that leads to RA. Molecules of bacterial or viral origin have been found in the joints of patients with RA (5, 8, 9), where they can trigger inflammatory reactions through pattern recognition receptors (PRRs).

PRRs are non-specific “sensors” of pathogen-associated (PAMPs) or damage-associated molecular patterns (DAMPs), playing a crucial role in modulating the activity of the innate arm of the immune response (10, 11). Different classes of PRRs have been characterized: the most studied being the Toll-like receptors (TLRs) and the nucleotide oligomerization domain-like receptors (NLRs) (10, 11). Our group focused its attention over the years on the expression and functions of a specific class of PRR, namely formyl peptide receptors (FPRs) (12). FPRs, like many other PRRs, are constitutively expressed on several cell types, including immune and epithelial cells (12). They sustain immune cell recruitment and activation (12) and regulate wound healing and homeostasis of epithelia (13–16).

A key function in modulating the inflammatory response has been defined for FPRs: they are classically able to sustain the inflammatory response, but, as a function of the context and/or the agonist, they can intervene in the resolution of the inflammatory response (17–19). This activity seems to be common to other PRRs (20, 21). This key role of FPRs in modulating the induction, the amplification, and the following physiologic resolution phase of the inflammatory responses prompted some research groups to study FPRs role in diseases whose pathogenesis is strictly linked to a strong imbalance between the inflammation and its resolution. TLRs and NLRs have already been defined as important for the pathogenesis of ankylosing spondylitis, psoriatic arthritis, systemic lupus erythematosus (SLE), RA, osteoarthritis (OA), and gout (22).

In the present review, we will focus on the data available in the literature on the role of FPRs and their ligands in RA, and we will discuss the results obtained querying a publicly available database collecting data from 90 RA patients with different clinical features (23).

## Formyl Peptide Receptors

FPRs are a group of G protein-coupled (GPCRs) chemoattractant receptors with an important role in host defense and inflammatory response (24). The *FPR* gene family can vary significantly in different mammalian species: the FPRs family includes FPR1, FPR2, and FPR3 in humans, and mFPR1, mFPR2/3, mFPR-rs1, mFPR-rs3, mFPR-rs4, mFPR-rs5, mFPR-rs6, and mFPR-rs7 in mice (25). The three genes encoding receptors mFPR1, mFPR2, and mFpr-rs1 are the best characterized. Although the complex evolution of the FPR gene family caused a high divergence between species orthologs, FPR1 is considered the mouse ortholog of human FPR1. Mouse FPR2 is a low-affinity receptor for N-formyl-methionyl-leucyl-phenylalanine (fMLF) and can be activated by several agonists of human FPR2 and FPR3. Further studies also indicate that mouse Fpr-rs1 share pharmacologic properties with human FPR2. The biological functions of other mouse *FPR* gene family members have not been clearly determined (25).

FPRs are mainly expressed in several types of innate immune cells, including neutrophils and monocytes/macrophages. In detail, macrophages express all three receptors (26, 27); neutrophils, monocytes, and natural killer cells express FPR1 and FPR2, but not FPR3 (26, 28); immature DCs express FPR1 and FPR3, while mature DCs express FPR3, but not FPR1 and FPR2 (29). The activation of FPRs in these cells induces chemotactic migration, phagocytic activity, and reactive oxygen species (ROS) production, mediating innate defense activity (25, 30). FPRs expression has also been reported in adaptive immune cells such as native CD4 T cells, human tonsillar follicular helper T cells, Th1 cells, Th2 cells, and Th17 cells (31).

Non-immune cells also express FPRs. For example, FPR1 is found in astrocytes, microglial cells, hepatocytes, and lung cells (32). FPR2 is the more ubiquitously expressed of the group, and it is found in synovial fibroblasts (33, 34), keratinocytes (35), brain cells, hepatocytes, microvascular endothelial cells (24), endocrine glands, intestinal epithelial cells (36, 37) and human bone marrow-derived mesenchymal stem cells (38–40). FPR3 is the least well-known of the three receptors, and its biological role has not been completely elucidated. This receptor is mainly expressed on monocytes and DCs, and it is located in intracellular vesicles rather than on the cell surface like the other FPRs (28, 41).

Our group described FPRs expression on basophils (42), gastric (16), and nasal (43) epithelial cells, and on fibroblasts (44).

FPRs, especially FPR1 and FPR2, have been shown to play a role in the development of several pathological conditions, such as neoplasms and inflammatory diseases. FPRs may act differently in these processes, both promoting and suppressing the disease progression. For example, FPR1 has a dual role in cancer development, playing a promoting role in glioblastoma (45, 46) and, conversely, tumor-suppressing functions in gastrointestinal cancers (19, 37, 47).

**Figure 1:**
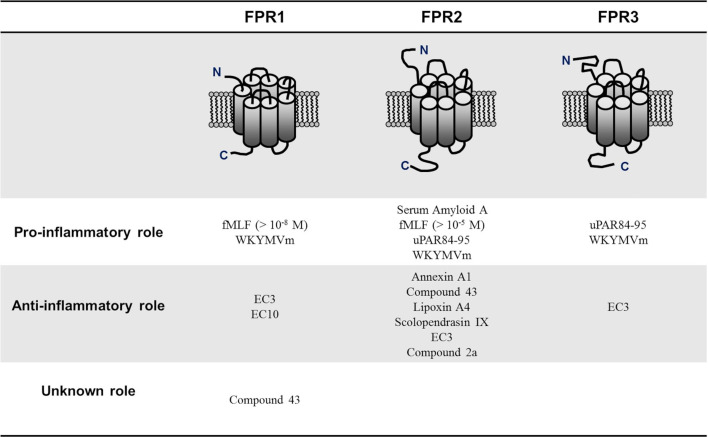
Pro-inflammatory and anti-inflammatory N-formyl peptide receptors (FPRs) ligands in rheumatoid arthritis. FPRs respond to various ligands with diverse classifications. Although most of FPRs ligands are involved in the clearance of infections, mediating chemotactic migration and phagocytic activity, other ligands activate pro-resolving, anti-inflammatory pathways. N-formyl-methionyl-leucyl-phenylalanine (fMLF); Trp-Lys-Met-Val-D-Met hexapeptide (WKYMVm), pyridazin-3(2H)-one derivative EC3 (EC3), pyridazin-3(2H)-one derivative EC10 (EC10); urokinase plasminogen activator receptor 84-95 (uPAR84–95).

Contradictory findings have also been observed dealing with the relationship between FPRs activation and infection response. For example, constitutively active FPRs were indispensable in the defense against the formation of biofilms by *Candida albicans* and aggressive infiltration by *Vibrio harveyi* (48, 49). Further studies are needed to elucidate this complex and apparently contradictory role to identify the different factors influencing FPRs behavior. However, one of the elements that may explain FPRs protean activity is that FPRs respond to various ligands with diverse classifications. Although most FPRs ligands are involved in the clearance of infections, mediating chemotactic migration and phagocytic activity, other ligands activate pro-resolving, anti-inflammatory pathways (24, 49). This duality in modulating inflammatory mechanisms is better expressed by FPR2, depending on ligand-specific conformational changes resulting in the switch between FPR2-mediated pro- and anti-inflammatory cell responses. In detail, it has been suggested that the binding of anti-inflammatory ligands such as Annexin A1 (AnxA1) caused FPRs to form homodimers, which led to the release of inflammation-resolving cytokines like IL-10; conversely, inflammatory ligands such as serum-amyloid alpha (SAA) did not cause receptor homodimerization (50). Generally, bacterial and mitochondrial formylated peptides are among those that classically activate a proinflammatory cell response, while AnxA1 and Lipoxin A4 (LXA4) are some of the better-known anti-inflammatory FPR2 ligands (49, 51). Many of these FPR2 ligands have also been suggested to play a promoting or protective role in RA. For example, SAA may induce several proinflammatory cytokines such as TNFα, IL-1β, IL-6, and matrix metalloproteinases-1 and -3, suggesting a role through the interaction with FPR2 in bone and cartilage destruction observed in RA (52). In turn, other FPR2 ligands such as AnxA1, LXA4, and Compound 43 (Cpd43) seem to exert a protective role in RA (**Figure 1**).

Through the years, several reports investigated the role of the FPRs or of specific members of the receptor family in the pathogenesis of RA. Gripentrog and coll. tried to establish the correlation between different FPR haplotypes and the pathogenesis of RA by analyzing 74 Caucasian RA patients and 74 controls. Although a specific FPR haplotype (i.e., 16A) was found only in the RA population, the authors had to conclude that only minor differences in haplotype distributions could be observed. It has to be taken into account, the low numbers of samples analyzed prevented from obtaining any conclusions regarding RA association to FPRs due to the lack of statistical power (53).

Other studies were conducted by evaluating the effects of receptor knock-out in mice models of arthritis or investigating the therapeutic effects of different FPR-agonists. The conclusion should consider the different experimental models used since several protocols are available to induce arthritis, but each involves different predominant mechanisms sustaining the joint inflammation and damage. We will in detail present the data published, presenting the evidence obtained by analyzing a specific component of the receptor/ligand system.

## FPRs and Their Ligands in RA Pathogenesis

Few groups investigated the role of FPRs in the modulation of chronic inflammatory conditions underlying RA pathogenesis. Indeed, FPRs are essential in the activation of inflammation but are also fundamental in its resolution. Most studies focused on the potential therapeutic effects of FPR2 activation mediated by its anti-inflammatory agonists. No direct data are available in the literature on the FPR1 role in RA.

### Formyl Peptide Receptor 2

Mice deficient in FPR2/3, the homologous to human FPR2, was used as a key model to address the role of FPR2 in the pathogenesis of arthritis (17). K/BxN serum transfer model in C57BL/6 mice induced arthritis by transfer of autoantibodies to glucose-6-phosphate isomerize. This model allows the generation of a synovial inflammation involving the participation of macrophages and neutrophils and the production of IL-1 and TNFα. FPR2^-/-^ mice displayed an exacerbation of arthritis symptoms following K/BxN serum transfer (17), supporting the evidence that, in particular, FPR2 could mediate anti-inflammatory effects (54) that could control RA pathogenesis.

### Annexin A1

Several reports regarding the protective role of FPRs in RA have been focused on the role of its ligand, AnxA1. AnxA1 is an endogenous anti-inflammatory mediator, exerting its inflammation-resolution functions by interacting with FPR2 (55).

AnxA1 has been found to be expressed in human RA synovial tissue (56–58) and has been identified as an important endogenous anti-inflammatory mediator in several animal models of RA (59).

AnxA1 and FPR2, but not FPR1, are in particular expressed by fibroblast-like synoviocytes (FLS) (54), the major cells promoting RA. It has been demonstrated that AnxA1 and FPR2 reduced FLS proliferation in an ERK and NF-kB-dependent manner and suppressed proinflammatory cytokine production from FLS (54).

In a different study, Dufton and coll. examined the effect of AnxA1 on T cell activation and differentiation and its implications for RA development, demonstrating that AnxA1 increases T cell activation in a Th1 sense. In collagen-induced arthritis (CIA) model, they administered AnxA1 to mice immediately after immunization with collagen for 12 days to evaluate the effects of AnxA1 in the early phase of RA development in which the Th1 phenotype is critical. These experiments showed that AnxA1 could increase arthritis symptoms if administered during the immunization phase of the CIA (60). Furthermore, an analysis of AnxA1 expression in T cells from RA patients and controls revealed higher protein expression levels in patients with RA than controls (60).

The authors discuss that the results mentioned above are only in apparent contrast with that obtained in AnxA1 null mice displaying an increased arthritic response. This could be due to the different etiology of joint damage and the different kinetics of the two models employed (CIA here, and antigen-induced arthritis in AnxA1 null mice) (54, 60). In the antigen-induced arthritis model, the Met-BSA induced arthritis through a Th2-response, demonstrating a protective AnxA1 role in RA (61). In the CIA model, a Th2-response reduction was described in the absence of Anx-A1, being this consistent with a Th2 pathogenic role in RA. The authors suggest to re-derivate the Anx-A1-null mice on the appropriate background to clearly define the role of Anx-A1 in the CIA model (60).

### Lipoxin A4

Lipoxin A4 (LXA4) is an endogenous lipoxygenase-derived mediator produced from arachidonic acid and exerting potent anti-inflammatory and pro-resolving effects on various cell types by activating FPR2 (62). Lipoxin A4 has been shown to suppress FLS production of proinflammatory cytokines and reduce RA severity in a CIA model (63).

### Compound 43

Cpd43 is a low molecular weight compound acting as an agonist for FPR2, although it has been reported to interact also with FPR1. Cpd43 exerts anti-arthritic effects in a model of K/BxN serum transfer. In particular, Cpd43 was demonstrated to be able to i) suppress TNFα expression in the joint; ii) inhibit osteoclast differentiation; iii) inhibit cytokine production in human FLS and macrophages in culture (54). Blocking FPR2, but not FPR1, abolished Cpd43 effects supporting the evidence that its protective role in the RA model is due to FPR2 (54).

A different study presents the results of Cpd43 administration to mice with CIA or antigen-induced arthritis (AIA). Cpd43 was able to reduce arthritis severity in both models: in CIA, Cpd43 decreased CD4 T cell proliferation and survival; in AIA, it increased CD4 T cell apoptosis. While inhibiting CD4 Th2 T cell proliferation and activity, Cpd43 was also able to increase the proportion of protective regulatory T cells (64). Furthermore, in both models, Cpd43 decreased TNF-sustained FLS proliferation (64).

### Scolopendrasin IX

An antimicrobial peptide - scolopendrasin IX - was identified from *Scolopendra subspinipes mutilans* used in the oriental medicine as a remedy for RA. This peptide acts as an agonist to FPR2 and showed therapeutic effects in RA by inhibiting cytokine production and neutrophil recruitment into the joint.

The administration of scolopendrasin IX in K/BxN serum-injected mice significantly decreased paw thickness, the clinical score of inflammatory arthritis, and markedly ameliorated joint destruction. The results obtained by Park et al. suggest that scolopendrasin IX was effective against inflammatory arthritis by blocking joint destruction (65). Scolopendrasin IX administration was also demonstrated to inhibit neutrophils recruitment into the synovium and their activation mediation by LPS (65).

### FPRs Agonists With Pyridinone and Pyrimidindione Scaffolds

Dr. Crocetti et al. identified three compounds with pyridinone and pyrimidindione scaffolds able to bind and activate, although with different affinities, the FPR family members. The pyridazin-3(2H)-one derivative EC3 (EC3) is a mixed FPR1/FPR2/FPR3 agonist; the pyridazin-3(2H)-one derivative EC10 (EC10) acts as an agonist to FPR1; and compound 2a is the most potent ligand identified with a 10-fold preference for FPR2 (66). The authors evaluated the therapeutic activity of the three compounds using a rat model of RA. All three compounds ameliorated the clinic of RA by increasing the pain threshold and reducing pain hypersensitivity (66).

### Serum Amyloid A

As mentioned above, FPR2 in humans can mediate both pro- and anti-inflammatory signals depending on the specific ligand (25). Among the pro-inflammatory FPR2 agonist, the role of Serum Amyloid A (SAA) in synovial damage has been investigated (33, 52, 67). It has been demonstrated that FLS, endothelial cells, and macrophages isolated from the synovial tissue of patients with RA patients expressed increased levels of SAA and FPR2 (52). In culture, SAA sustains FLS proliferation and survival (33), stimulates metalloproteases production by FLS (52), stimulates the proliferation, migration, and tube formation of endothelial cells (33). Finally, SAA induces in rheumatoid synoviocytes the expression of Pentraxin 3 (PTX3), an acute-phase reactant involved in amplifying the inflammatory response (67). This evidence, taken together, sustains the pathogenic role of SAA in RA.

### FPRs Agonists fMLF, uPAR84–95, and WKYMVm Peptide

We have recently demonstrated (44) that fibroblasts obtained from skin biopsies of patients affected by Systemic Sclerosis (SSc) express all three receptors for the N-formyl peptides. The expression of these receptors was highly increased compared to normal skin fibroblasts both at mRNA and protein levels. In addition, we conducted experiments using specific agonists [i.e., fMLF, urokinase plasminogen activator receptor 84-95 (uPAR84–95), and Trp-Lys-Met-Val-D-Met hexapeptide (WKYMVm)], demonstrating that upon stimulation, SSc fibroblasts from affected subjects were able to proliferate, migrate, and transform into a myofibroblast phenotype as assessed by ROS generation, matrix deposition, and α-smooth muscle actin (α-SMA) overexpression as compared to normal skin fibroblasts. In order to evaluate whether FPRs stimulation plays a role in some ROS-mediated processes such as tissue remodeling and fibrosis, we then conducted experiments on BJ normal fibroblasts showing that FPRs stimulation led to Rac1 and ERKs activation, promoting gp91^phox^ and p67^phox^ expression as well as a direct interaction between GTP-Rac1 and p67^phox^ (68). However, the possible involvement of the FPRs in other more common autoimmune conditions such as RA has been only partially confirmed.

## Association of FPRs With Clinic-Pathologic Parameters of RA Patients

### Rheumatoid Arthritis Histopathotypes

Heterogeneity in the quality and quantity of the synovial cellular infiltrate is well recognized, and it has been evaluated as a possible biomarker of treatment response in patients with RA (69). Recently published data reporting cellular and molecular analyses of synovial tissue from a cohort of 144 patients with treatment-naïve early RA demonstrated for the first time the presence of three pathology groups: i) lympho-myeloid dominated by lymphoid lineage infiltration (T cells, B cells, plasma cells) in addition to myeloid cells; ii) a diffuse-myeloid group characterized by macrophage or monocyte enrichment, but poor in B cells/plasma cells; and iii) a pauci-immune fibroid group showing a distinct lack of immune-inflammatory infiltrate and prevalent stromal cells (70). They also demonstrated that synovial cellular and molecular signatures define prognostic and treatment phenotypes, such as the response to disease-modifying antirheumatic drug (DMARD) therapy, clinical outcome, and radiographic joint damage (70). Moreover, integrating histological and molecular signatures into a clinical prediction model may help predict whether patients will require biological therapy. For instance, recent data by Lliso-Ribera and colleagues suggest that the lympho-myeloid pathotype, with a dense synovial infiltrate enriched in B cells and significant upregulation of T/B cell genes at disease onset, predicted poor outcome with the need for biological therapy irrespective of clinical classification (71). This evidence is in line with recently published data in early RA that reports the association between the lympho-myeloid pathotype with highly aggressive disease and worse radiographic outcomes (70). The analysis of the synovial histopathology has also been evaluated as a helpful tool to identify among clinically indistinguishable patients those with a lower probability of response to TNFα-blockade (69), especially the pauci-immune pathotype could predict an inadequate response to treatment with TNFα antagonists. In a recent study, Lewis and colleagues (23) analyzed the histology and RNA-seq of synovial biopsies from a large cohort of early treatment-naïve patients [the Pathobiology of Early Arthritis Cohort (PEAC)]. From this larger cohort, they selected 90 individuals meeting the 1997 ACR classification criteria for early RA to identify the three histological pathotypes and reveal gene modules associated with RA severity and clinical outcome. They analyzed gene expression changes at the RNA sequencing level in both blood and synovium from the same RA patient and identified transcriptional endotypes in the synovium linked to the three distinct pathotypes. They also combined RNA-seq with detailed synovial histology and correlated these molecular signatures with clinical and imaging phenotype data at disease presentation. Finally, the authors developed a data exploration website (available at https://peac.hpc.qmul.ac.uk/) to dissect gene signatures across synovial and blood compartments, integrated with deep phenotypic profiling. Herein, we used the data exploration website developed by Lewis and colleagues (23) for describing the gene expression of FPRs in both blood and synovium in patients with early RA.

#### FPRs Are Differently Expressed in the Distinct Rheumatoid Arthritis Pathotypes

As shown by the RNA sequencing (RNA-seq) analysis of synovial biopsies and blood of patients data available from PEAC (http://www.peac-mrc.mds.qmul.ac.uk) (23), the three FPR receptors show a different expression in the distinct RA pathotypes: the fibroblastic pauci-immune pathotype, the macrophage-rich diffuse-myeloid pathotype, and the lympho-myeloid pathotype, suggesting different pathogenic pathways or activation disease states.

FPR1-lymphoid expression at synovial level was greater in comparison to the expression in the other pathotypes, while at blood level, FPR1 expression showed similar mean values in lymphoid, myeloid, and fibroid subgroups. In addition, the mean gene expression of FPR1 was greater in blood than that observed in the synovial samples

FPR2 expression showed an opposite pattern compared to that of FPR1. Indeed, at the synovium level, FPR2 genes were less expressed in all the pathotypes as compared to blood expression (**Table 1**). Moreover, the mean gene expression was markedly lower in synovial samples of all pathotypes than the mean expression of FPR1 (**Table 1**).

**Table 1:** FPRs mean gene expression in synovial and blood samples.

	FPR1		FPR2		FPR3	
	Synovial	Blood	Synovial	Blood	Synovial	Blood
Lymphoid	11.23	14.47	6.94	12.53	12.28	5.12
Myeloid	10.75	14.52	6.60	12.59	11.92	4.89
Fibroid	9.88	14.18	6.61	12.35	11.22	5.17

Adapted from http://www.peac-mrc.mds.qmul.ac.uk.

FPR3 mean gene expression was higher in comparison to FPR1 and FPR2 (**Table 1**), and it was upregulated in the lympho-myeloid pathotype.

**Figure 2:**
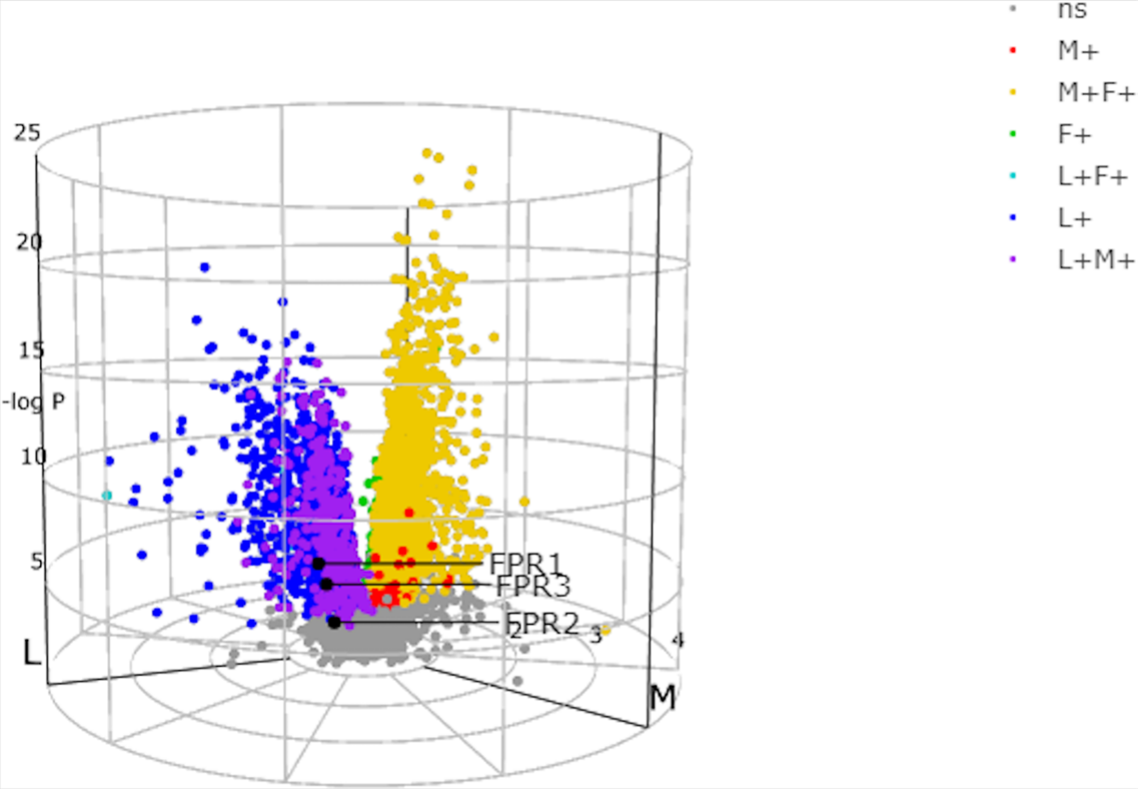
N-formyl peptide receptors (FPRs) expression in the three histologically identified subgroups using the 3D volcano plot. In the three-way volcano plots genes, which were significantly upregulated in one group alone, were colored using primary colors, blue in the lympho-myeloid group (L+), red in the diffuse-myeloid group (M+), and green in the pauci-immune fibroid (F+). Moreover, genes upregulated in two groups (compared to the minimum reference group) were illustrated using secondary colors, i.e., genes upregulated in lympho-myeloid and diffuse-myeloid compared to pauci-immune fibroid: purple; upregulated in diffuse-myeloid and pauci-immune fibroid versus lympho-myeloid: yellow; upregulated in lympho-myeloid and pauci-immune fibroid versus diffuse-myeloid: cyan. Non-significant genes (ns) are colored gray. FPR1 and FPR3 are colored in blue, FPR2 gene is colored in gray. Adapted from http://www.peac-mrc.mds.qmul.ac.uk.

The different expression of the FPRs genes in the three histologically identified subgroups can be visualized though the 3D volcano plot (**Figure 2**). FPR1 gene using the 3D volcano plot was depicted in blue, showing that this receptor was upregulated in the lympho-myeloid pathotype and the fold change was significant compared to the other groups (r=0.828 p=5.99^-6^). FPR2 was depicted in grey, demonstrating a not significant difference in the expression in the pathotypes. FPR3 was upregulated in the lympho-myeloid, as confirmed by the primary color blue, which identifies the lympho-myeloid group. The fold change, used as an alternative to the Z score (indicating the vectors for pathotype per gene), showed that the upregulation of FPR3 was significant compared to the other groups (r=0.979, p=3.71^-5^).

**Figure 3:**
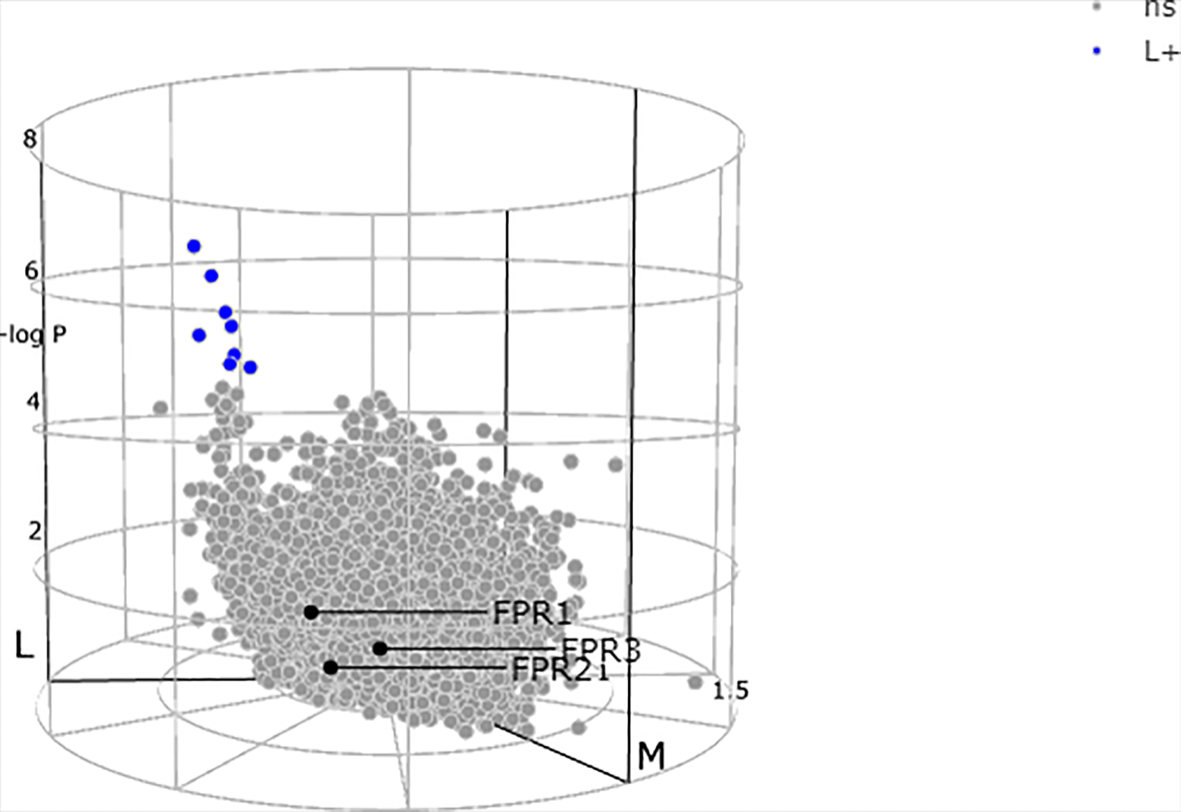
N-formyl peptide receptors (FPRs) expression at blood level showing a non-significant (ns) expression between the subgroups as demonstrated by all the three genes colored in gray. Adapted from http://www.peac-mrc.mds.qmul.ac.uk.

Lewis and colleagues (23) demonstrated a stark difference in the absolute quantity of differentially expressed transcripts among the pathotypes, with nearly 3,000 transcripts in synovium compared to only 8 differentially expressed transcripts in corresponding peripheral blood. All the three receptors FPRs, at blood level, showed a non-significant expression between the subgroups as demonstrated by the genes colored in grey (**Figure 3**) (23, 70, 72).

## Concluding Remarks and Future Perspectives

The currently available literature concerning the relationship between FPR1, FPR3, and RA is scarce. By analogy to the role played in other autoimmune diseases such as SSc (44), we can suppose that FPR1 exerts a potentially proinflammatory role in RA. However, very little work has been done to further explore this connection. Data related to FPR3 have been only partially evaluated since FPR3 is the least well-known of the three receptors, and its biological role has not been completely elucidated. FPR2 is the more ubiquitously expressed of the FPRs (26). Given its potentially protective role in RA, the interest in FPR2 and its ligands has recently grown. Indeed, a better understanding of the complex interaction between FPR2 and its ligands may help establish these molecules as potential therapeutic interventions.

In this review, we have widely discussed the potential effects and roles of FPRs ligands in different pathological models. However, this study has some limitations. Data related we extrapolated querying the online database developed by Lewis and colleagues (23), referred only to the gene expression of FPRs in both blood and synovium in patients with early RA. No comparison on the healthy subject’s basal levels of FPRs can be made. In addition, data from the interactive website (https://peac.hpc.qmul.ac.uk/) report synovial, and blood gene expression, but no data are available on protein expression.

In synovial biopsies that FPR1 and FPR3 were significantly increased in all pathotypes, whereas FPR2 showed an opposite pattern of expression, being less represented. The lower FPR2 gene expression in the RA cohort patients could be related to the protective role played by this receptor in the disease pathology. Indeed, several authors described that among the three members of the N-formyl peptide receptor family, FPR2 could mediate anti-inflammatory effects (54), playing a role in the pathogenesis of RA. Moreover, AnxA1, expressed in human RA synovial tissue (56–58), has been recognized as a significant endogenous anti-inflammatory mediator in several animal models of RA.

Conversely, FPR1 is expressed at a high level in the lympho-myeloid subgroup, which is related to a high disease activity measured by DAS28-ESR/CRP and confirmed by an aggressive radiological and radiographic involvement, causing a poor clinical and therapeutic outcome.

In conclusion, FPRs are characterized by multifaceted roles that encourage researchers to target these receptors to treat several inflammatory and neoplastic diseases.

## Author Contributions 

All authors contributed to the article and approved the submitted version. IM, NP, and FR participated in planning the study, analysis and interpretation of data, drafting the article, critical revision of the article for important intellectual content, final approval of the article. FG, VP, and AP participated in drafting the article, critical revision of the article for important intellectual content, final approval of the article.

## Funding

The authors declare that this study received funding from Orpha Biotech. The funder was not involved in the study design, collection, analysis, interpretation of data, the writing of this article or the decision to submit it for publication.

## Conflict of Interest

The authors declare that the research was conducted in the absence of any commercial or financial relationship that could be construed as a potential conflict of interest.
